# Flavonoids’ Effects on *Caenorhabditis elegans’* Longevity, Fat Accumulation, Stress Resistance and Gene Modulation Involve mTOR, SKN-1 and DAF-16

**DOI:** 10.3390/antiox10030438

**Published:** 2021-03-12

**Authors:** María Alejandra Guerrero-Rubio, Samanta Hernández-García, Francisco García-Carmona, Fernando Gandía-Herrero

**Affiliations:** Unidad Docente de Biología, Departamento de Bioquímica y Biología Molecular A, Facultad de Veterinaria, Regional Campus of International Excellence “Campus Mare Nostrum’’, Universidad de Murcia, 30100 Murcia, Spain; mariaalejandra.guerrero@um.es (M.A.G.-R.); samanta.hernandez@um.es (S.H.-G.); gcarmona@um.es (F.G.-C.)

**Keywords:** *Caenorhabditis elegans*, DAF-16/FOXO, flavonoids, mTOR, PARPs, SKN-1/Nrf2

## Abstract

Flavonoids are potential nutraceutical compounds present in diary food. They are considered health-promoting compounds and promising drugs for different diseases, such as neurological and inflammatory diseases, diabetes and cancer. Therefore, toxicological and mechanistic studies should be done to assert the biological effects and identify the molecular targets of these compounds. In this work we describe the effects of six structurally-related flavonoids—baicalein, chrysin, scutellarein, 6-hydroxyflavone, 6,7-dihydroxyflavone and 7,8-dihydroxyflavone—on *Caenorhabditis elegans’* lifespan and stress resistance. The results showed that chrysin, 6-hydroxyflavone and baicalein prolonged *C. elegans’* lifespan by up to 8.5%, 11.8% and 18.6%, respectively. The lifespan extensions caused by these flavonoids are dependent on different signaling pathways. The results suggested that chrysin’s effects are dependent on the insulin signaling pathway via DAF-16/FOXO. Baicalein and 6-hydroxyflavone’s effects are dependent on the SKN-1/Nfr2 pathway. In addition, microarray analysis showed that baicalein downregulates important age-related genes, such as mTOR and PARP.

## 1. Introduction

Flavonoids are low-molecular-weight polyphenols present in plants that have been consumed for ages, as they are found in several foods of plant origin, such as cocoa, tea, fruits and wine. Currently, around 8000 flavonoid-like structures have been identified [1]. They appear as secondary metabolites in vegetables and have different roles in development, in defense against plagues or stress, in protection from UV light and also as pigments in most of the angiosperm families. Structurally, there are two main groups, the 2-phenylchromans that are strictly flavonoids, including flavanones, flavones, flavonols, flavan-3-ols and anthocyanidins; and the 3-phenylchromans that are isoflavonoids, such as isoflavones, isoflavans and pterocarpans [2]. Flavonoids are considered health-promoting and disease-preventing compounds, since they are associated with several positive effects related to their antioxidant, anti-inflammatory, anti-mutagenic and anti-carcinogenic activities [3]. They are also potent inhibitors of some key enzymes, such as cyclo-oxygenase (COX) [4] and lipoxygenase (LOX) [5] and possible candidates in neurodegenerative disease treatments (Alzheimer’s and Parkinson’s treatments) [6,7].

**Figure 1:**
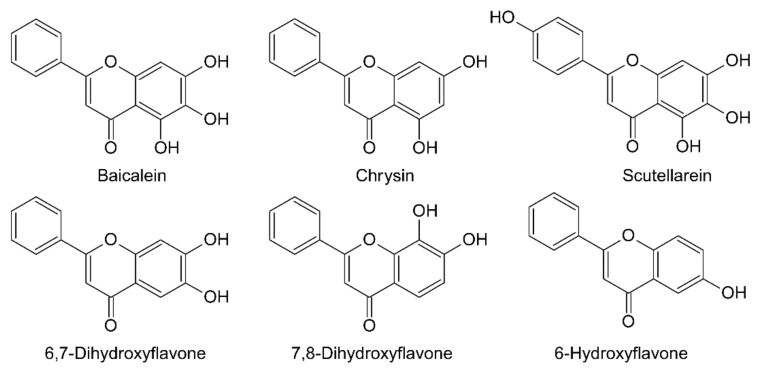
Chemical structures of the flavonoids used in this work.

Their potential as nutraceuticals and pharmaceutical compounds for the prevention of age-related diseases encouraged us to investigate the effects of six structurally related flavonoids in the animal model *Caenorhabditis elegans*. In this work, six structurally related flavonoids were chosen: baicalein, chrysin, scutellarein, 6-hydroxyflavone, 6,7-dihydroxyflavone and 7,8-dihydroxyflavone (Figure 1 and Table S1). Several reports have studied the effects of these molecules as antioxidant and radical scavengers, and their effects in cell lines [8,9]; however, there are few studies of *C. elegans* related to flavones, and they are mainly focused on baicalein [10]. Other flavonoids such as myricetin, quercetin and kaempferol’s effects on *C. elegans’* lifespan and ROS-levels have previously been reported [11], but the studies did not elucidate the molecular mechanisms underlying the positive effects described.

The health-promoting effects of baicalein, chrysin, scutellarein, 6-hydroxyflavone, 6,7-dihydroxyflavone and 7,8-dihydroxyflavone have been investigated in the animal model *C. elegans*. In addition, the molecular mechanisms of action for the six flavones have been investigated by using *C. elegans* mutant strains and microarray assays.

## 2. Materials and Methods

### 2.1. Chemicals

The tested flavonoids—baicalein, scutellarein, chrysin, 6-hydroxyflavone, 6,7-dihydroxyflavone and 7,8-dihydroxyflavone—were purchased from Extrasynthese (Genay, Cedex, France). Working solutions of each flavonoid were prepared at 25 mM in DMSO (dimethyl sulfoxide) and kept at −20 °C until use. In the experiments, DMSO was used as control and was maintained at a fixed concentration of 0.4%. The rest of the reagents were obtained from Sigma-Aldrich (St. Louis, MO, USA). Double distilled water (d.d. water) was obtained using a Milli-Q system (Millipore, Bedford, MA, USA).

### 2.2. Caenorhabditis elegans Strains and Culture Conditions

Caenorhabditis Genetic Center (CGC, St. Paul, MN, USA), which is funded by NIH Office of Research Infrastructure Programs (P40 OD010440), provided us with the strains used in this study, i.e., the wild type N2, TJ375 (*gpIs1 [hsp-16.2p::GFP]*), CF1038 (*daf-16(mu86)*), LD1 (*ldIs7 [skn-1b/c::GFP* + *rol-6(su1006)]*), QV225 (*skn-1(zj15)*), TJ356 (*zIs356 [daf-16p::daf-16a/b::GFP* + *rol-6(su1006)*]), CL2166 (*dvIs19 [(pAF15)gst-4p::GFP::NLS] III*) and LD1171 (*ldIs3 [gcs-1p::GFP + rol-6(su1006)]*). Worms were maintained at 20 °C in solid nematode growth medium (NGM) [12]. The *Escherichia coli* strain OP50, employed as a food source, was grown overnight in LB (Luria–Bertani) medium at 37 °C and then concentrated 10× in sterile M9 buffer. This work has received approval for research ethics from “Committee on Animal Research and Ethics” (CEEA, code 371/2017, date 19 July 2017), University of Murcia (Spain).

### 2.3. Synchronous Worm Culture

All the worms used in this study were age synchronized. Synchronic cultures of *C. elegans* were obtained using the bleaching method as follows: adults from 3-day plates were collected by using 3.5 mL of sterile M9 buffer, and then mixed with 1.5 mL of freshly prepared household bleach/5N NaOH solution (2:1). The solution was mixed by vortex every 2 min for 10 min at room temperature and then centrifuged at 7500× *g*. The supernatant was discarded and the pellet was washed twice with 5 mL of M9 buffer. A 10 mL solution of fresh M9 buffer was used to keep the eggs overnight at 20 °C and orbital shaking for their synchronous hatching.

### 2.4. Expression of HSP-16.2p::GFP in C. elegans TJ375

TJ375 (*hsp-16.2p::GFP*) nematodes were grown in liquid S medium at 20 °C and the expression of HSP-16.2 was measured by observing the fluorescence of the fused green fluorescent protein (GFP). The day after synchronous hatching, TJ375 worms were treated with 100 µM of each flavonoid. After 48 h, the worms were transferred to fresh S medium, where the naphthoquinone juglone was added at 20 μM to induce oxidative stress. After 24 h, worms were washed with fresh M9 buffer and seated onto glass slides which contained 10 mM sodium azide to avoid the worms’ mobility. For each condition, the fluorescence of 8–10 worms was observed by using the 20× lens and the I3 filter cube in a Leica DM 2500 LED microscope (Leica Microsystems, Wetzlar, Germany). Images were captured at constant exposure times using a Leica DFC550 camera fitted to the microscope. The images taken each showed the anterior part of a worm named the pharynx, which stretches from the mouth to the pharyngeal-intestinal valve. ImageJ software (NIH) was used to quantify GFP fluorescence [13] through black-to-white inversion of each image and subsequent measurement of mean pixel density.

### 2.5. C. elegans Survival Assays Monitored with the Lifespan Machine

Lifespan Machine, the platform for the automatic control of *C. elegans* mobility, was used in the survival assays. After 48 h in liquid S medium supplemented with the tested flavonoids, synchronic worms were centrifuged at 2000× *g* and washed with M9 buffer three times. Then, 40–50 worms were transferred to 35 mm analysis plates, containing 8 mL of NGM agar, nystatin 30 µg/mL to avoid mold contamination, ampicillin 100 µM to avoid bacterial contamination and supplemented with 10 µg/mL of FUdR (2′-Deoxy-5-fluorouridine) to avoid progeny [14] and 100 µL of heat inactivated *E. coli* OP50 as food source (a culture grown overnight in LB at 37 °C was concentrated 10× in sterile M9 buffer and then incubated 30 min at 65 °C for its inactivation). All the experiment plates were performed in triplicate. Four control plates were included in every scanner to counteract the possible lifespan variations due temperature oscillations caused by the disposition of the scanners and plates. Lifespan results of sample plates were only compared with the control plates placed in the same scanner. Plates were closed and incubated 20 min at 20 °C. Plates were placed face down (close lid included) into the Lifespan Machine’s scanners. Lifespan Machine was employed following the constructor instructions described by Stroustrup et al. 2013 [15] and the modifications detailed by Guerrero-Rubio et al. 2019 [16]. The experiments were set in the Lifespan Machine at 25 °C for 25 days.

### 2.6. Data Statistical Analysis

The results obtained in the Lifespan Machine were analyzed using OASIS 2, an on-line platform for mathematical analysis of survival data [17], with the Kaplan–Meier estimator, Boschloo’s test, Kolmogorov–Smirnov test and log-rank test. The Kaplan–Meier estimator was used to obtain the survival curves and the mean lifespan was calculated as the area under the survival curve. The log-rank test allowed the comparison of two survival functions through overall lifespan data, the differences in the survival data were significant if *p*-value ≤ 0.05. One-way ANOVA Calculator for independent measures was used for numeric data (fluorescence measurements, and ORO measurements). For nominal data, Chi-square Calculator Contingency Table was used (intracellular localization of *daf-16* and *skn-1*). All these analyses were performed using the online Social Sciences Calculator https://www.socscistatistics.com (accessed on 10 February 2021). Two independent trials were performed for the different experiment, with a number of individuals (*n*) ≥ 10. The significance level for all the data was established in 0.05.

### 2.7. Intracellular Localization of the Transcription Factor DAF-16

TJ356 (*daf-16::GFP*) worms express green fluorescent protein joined to the transcription factor DAF-16. TJ356 nematodes were age-synchronized and treated with 100 μM of each flavonoid. After 72 h at 20 °C, they were washed twice with M9 buffer and settled onto glass slides containing 10 mM sodium azide. Images were taken with the above-mentioned microscope and under the same conditions (20× lens, I3 filtercube). Worms were classified as “nuclear” whether GFP fluorescence was detected in the nuclei, “cytoplasmic” whether GFP fluorescence was present in the whole body and “intermediate” whether fluorescence was detected both into nuclei and cytoplasm. Non-treated TJ356 worms were heat shocked for one hour at 37 °C and then employed as positive control (nuclear localization). Non-treated worms kept in water were used as negative control (cytoplasmic localization).

### 2.8. Intracellular Localization of the Transcription Factor SKN-1

The strain LD1 expresses skn-1b/c::GFP in the gut and in the ASI neurons. SKN-1 is involved in Phase II detoxification where it acts by regulating a key gene through constitutive and stress-inducible mechanisms in the ASI chemosensory neurons and intestine, respectively. Thus, SKN-1 is detected in ASI nuclei in normal conditions, but it is accumulated in intestinal nuclei in response to oxidative stress. Age-synchronized LD1 worms were treated with 100 µM of each flavonoid in S medium. After 72 h at 20 °C, the nematodes were washed twice with M9 buffer and deposited onto glass slides containing 10 mM sodium azide. Water was used as negative control. Images were taken with the above-mentioned microscope and under the same conditions (20× lens, I3 filtercube). ImageJ software (NIH) was used to measure gut fluorescence of each image which were previously split in red, blue and green channels. Only fluorescence intensity detected in the green channel was employed for quantification of SKN-1 in the gut area under the pharynx.

### 2.9. Flavonoids Effect on GST-4p and GCS-1p Expression

Synchronous animals of the CL2166 and LD1171 strains were treated with 100 μM of each flavonoid or DMSO for 72 h at 20 °C in S medium, as positive control juglone 20 μM was used. The animals were visualized under fluorescence microscope at 20× lens with the I3 filter cube. The image acquisition and analysis were performed as in the Section 2.8.

### 2.10. RNA Extraction

Age-synchronized nematodes were treated with 100 μM of baicalein in S medium or DMSO as control the day after hatching. After 48 h at 20 °C and orbital shaking, worms were washed with M9 buffer ten times for the complete discard of *E. coli* present in the medium. Then, 250 μL of M9 buffer were employed to resuspend the washed worms and 750 μL of TRIzol^®^ was added for the RNA extraction. RNA extraction and its purification were performed following the specifications described for PureLinkTM RNA Mini Kit from Invitrogen (Carlsbad, CA, USA). The amount and quality of the RNA obtained were measured by Bioanalyzer (Agilent Technologies, Santa Clara, CA, USA) and then used for microarray analysis.

### 2.11. Microarray Analysis

3.5 ng of each sample were used to synthetize Ss-cDNA through the GeneChip WT Pico Reagent kit (Affymetrix, P/N 703262) obtained from Thermo Fisher Scientific (Thermo Fisher Scientific Inc., Waltham, MA, USA). The protocol supplied by the manufacturer was followed and the amount and quality of ss-cDNA were checked by Nanodrop and Bioanalyzer. ss-cDNA targets were cleaned up and after fragmentation and terminal labelling, 3.75 µg of fragmented and biotinylated ss-cDNA were included in the hybridization mix, using the GenAtlas Hybridization, Wash and Stain kit for WT Array Strips (Affymetrix, P/N 901667, Affymetrix Inc., Santa Clara, MA, USA) following manufacturer’s protocol. The resulting preparations were hybridized to GeneChip^®^ *C. elegans* Gene 1.1 ST Array Strip (Affymetrix, 902157, Affrymetrix Inc., Santa Clara, MA, USA) which contains 26 unique sequences of each transcript. Preliminary hybridization tests (peak controls) and labeling were performed to ensure that the chips passed the quality criteria. Then, Affymetrix Expression Command Console (Affymetrix Inc., Santa Clara, MA, USA) was used to process microarrays data and to detect that all samples were within bounds for hybridization and labeling tests.

Three independent samples were used to perform the experiment in triplicate. Samples from worms treated with baicalein were grouped as “treatment” and worms treated with DMSO instead of baicalein were grouped as “DMSO control.” RMA (Robust Multiarray Average) was used to perform data analysis by allowing that raw intensity values were background corrected, log2 transformed and then quantile normalized in order to obtain an individual intensity value for each probe set. Partek Genomics Suite and Partek Pathways software (Partek Incorporated, St. Louis, MO, USA) were used for statistical analysis and an ANOVA test was applied with a restrictive threshold at *p*-value ≤ 0.05. The molecular interaction, reaction and relation networks that showed differentially expressed genes (DEGs) were then analyzed using KEGG Pathways Kyoto Encyclopedia of Genes and Genomes.

### 2.12. Oil Red O (ORO) Lipid Staining

Quantification of lipids was performed using the Oil Red O protocol [18] with some modifications. Briefly, one day adult worms treated with different concentrations of baicalein (0, 10, 25, 50, 100 µM) were fixed in a formaldehyde PBS solution (4%) for 24 h at 4 °C. Then, the worms were washed and permeabilized using a 5% β-mercaptoethanol, 1% Triton-100 solution in tris/HCl buffer pH 7.4 for 24 h at 37 °C. Then the fixed and permeabilized worms were washed twice with PBS. Meanwhile, an ORO working solution (60%) was prepared diluting a stock solution of ORO (0.5 g ORO in 100 mL of isopropanol) in d.d. water and filtered through a 0.22 µm syringe filter. Fixed worms were treated with the ORO working solution for five minutes at room temperature, then washed three times with PBS-Triton (0.01%) buffer. ORO stained animals were mounted onto glass slides and brightfield images were taken with the 40× lens of a Leica DM 2500 LED microscope fitted with a Leica DFC550 camera (Leica Microsystems, Wetzlar, Germany). ORO quantification of the images was performed with the free software Fiji (NIH), an improved ImageJ [19]. Firstly, the images were split with the color deconvolution tool using the H AEC vector, followed by applying the thresholding tool only in the red channel and measuring the intensity in the threshold area.

## 3. Results

### 3.1. Effect of Flavonoids on C. elegans Oxidative Stress Resistance

**Figure 2:**
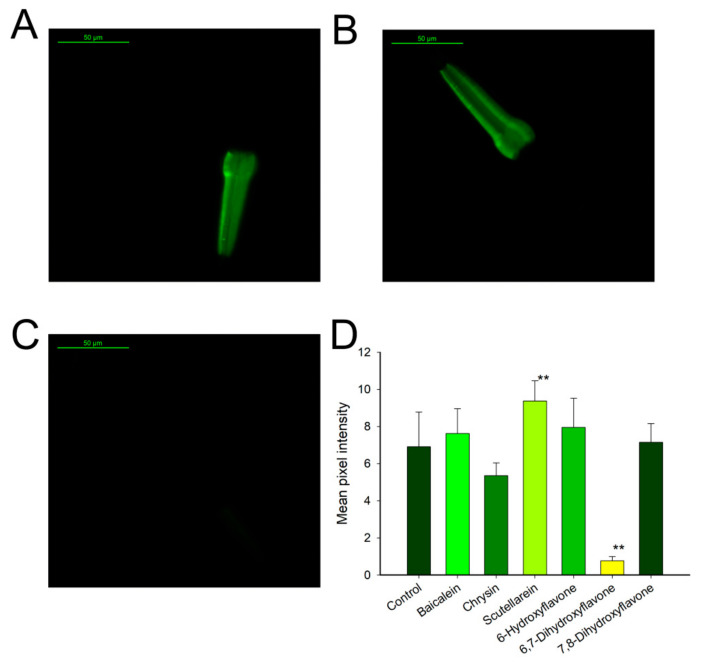
Flavonoids effect on *Caenorhabditis elegans* strain TJ375 (*hsp-16.2p::GFP*) model of oxidative stress resistance. (**A**) Representative image of a TJ375 control worm. (**B**) Representative image of a TJ375 worm treated with 100 µM of baicalein. (**C**) Representative image of a TJ375 worm treated with 100 µM of 6,7-dihydroxyflavone. (**D**) Quantification of the effect of pre-treatment with flavones in oxidative stress resistance measured through fluorescence integration. Data are represented as mean ± S.D, *n* > 10; two independent experiments were performed; ** significant at *p* ≤ 0.05 by ANOVA test. Scale bar: 50 μm, magnification 40×.

To estimate the ability of the six flavonoids to enhance the defense against oxidative stress, the TJ375 strain of *C. elegans* was fed with them individually, and then *C. elegans* was exposed to oxidative stress chemically induced by juglone (Figure 2A). This strain presented the HSP16.2 fused with the green fluorescent protein GFP, and it was located in the pharynx. In response to oxidative stress, the fused hsp-16.2p::GFP accumulated bright green fluorescence (Figure 2A). When TJ375 worms were treated with 100 µM of flavonoids, only 6,7-dihydroxyflavone and chrysin (Figure 2D) had any protective effect against the induced stress; 6,7-dihydroxyflavone presented the highest potential as an in vivo antioxidant with a decrease in fluorescence of 88.9%.

Additionally, strains LD1171 (*gcs-1p::GFP*) and CL2166 (*gst-4p::GFP*) were used to study the flavones effects on specific antioxidants and phase II class detoxification genes, including gamma-glutamine cysteine synthetase-1 (gcs-1) and glutathione S-transferase 4 (gst-4). GCS-1 catalyzes the rate-limiting first step in glutathione biosynthesis, while GST-4 has a role in the conjugation of electrophilic substrates to glutathione (GSH) [20]. Both enzymes offer protection against oxidative stress and promote longevity. Under normal conditions, *gst-4*::GFP is expressed primarily in the muscles and the hypodermis, and is increased in response to a variety of inductors (Figure S1A,C) [20]; treatment with 100 µM of 6-hydroxyflavone and 6,7-dihydroxyflavone increased its expression by 65.3% and 94.3% respectively. Meanwhile, neither scutellarein nor 7,8-dihydroxyflavone had a significant effect on the expression of GST-4. On the other hand, baicalein was the most effective on the upregulation of GCS-1 expression (73.5%), followed by 6-hydroxyflavone, 6,7-dihydroxyflavone and 7,8-dihydroxyflavone, which increased the expression by 55.3%, 51.4% and 45.4% respectively (Figure S1F). GCS-1 is expressed in *C. elegans* in the pharynx, ASI chemosensory neurons and anterior and posterior intestine; in Figure S1C, the expression in the pharynx and the ASI neurons is shown.

### 3.2. Effects of Flavonoids on Fat Accumulation in C. elegans

**Figure 3:**
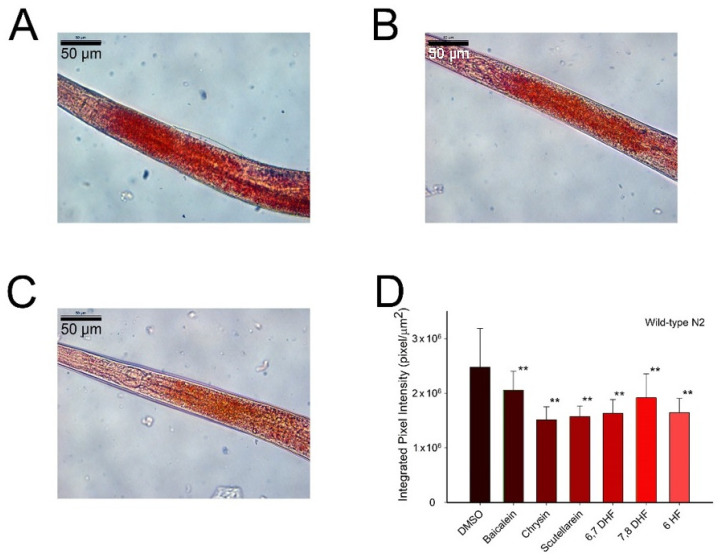
Effects of flavonoids on fat accumulation in *C. elegans* measured with Oil Red O (ORO). (**A**) DMSO control worms. (**B**) Worm treated with 100 µM of scutellarein. (**C**) Worm treated with 100 µM of chrysin. (**D**) Lipid content measurement with ORO staining in *C. elegans* treated with 100 µM of each flavonoid. Data are represented as mean ± S.D, *n* > 10; two independent experiments were performed; ** significant at *p* ≤ 0.05 by ANOVA test. Scale bar: 50 μm, magnification 40×.

Oxidative stress plays a key role in several metabolic diseases, such as insulin resistance and diabetes. Obesity and fat accumulation increase the oxidative stress damage, and on contrary, an increase in ROS production leads to an excessive accumulation of fat [21,22]. Therefore, the search for small molecules with antioxidant activity that are able to regulate fat metabolism may be useful to alleviate the effects of metabolic diseases. To evaluate the in vivo effect of flavonoids as fat mobilizers, the stored fat of *C. elegans* was measured in worms treated with 100 μM of flavonoids and in control worms using the Oil Red O (ORO) staining protocol (Figure 3 and Figure S2). The results showed that all the tested molecules were able to reduce the fat storage in the animals (Figure 3D). The most effective flavonoids for reducing the animal’s fat were chrysin and scutellarein, which resulted in 38.9% (Figure 3C,D) and 36.6% (Figure 3B,D) less fat than control worms, respectively.

### 3.3. Effect of Flavonoids on C. elegans Lifespan

**Figure 4:**
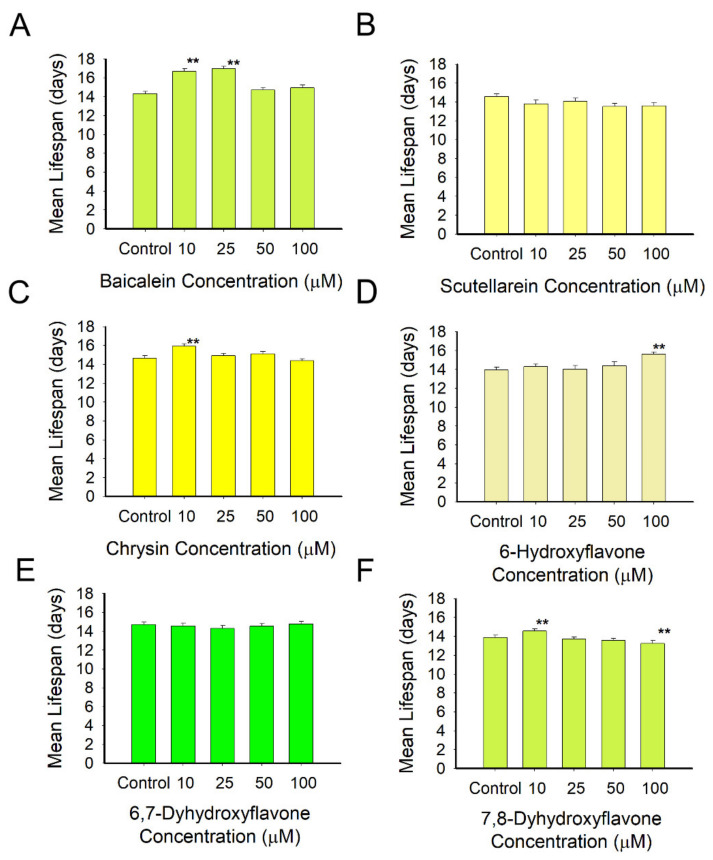
Mean lifespans of wild-type *C. elegans* treated with different doses of flavonoids (0, 10, 25, 50 and 100 µM). (**A**) Baicalein, (**B**) scutellarein, (**C**) chrysin, (**D**) 6-hydroxyflavone, (**E**) 6,7-dihydroxyflavone and (**F**) 7,8-dihydroxyflavone. Data are represented as mean lifespan ± S.E. ** significant at *p* ≤ 0.05 by log-rank test.

Lifespan is a useful physiological parameter to set up the effects of a compound in the animal model, either positive (longevity increase) or negative (toxicity). The Lifespan Machine was employed to measure the mean lifespan of the flavonoid-fed worms (Figure S3). The results showed that a dose of 10 µM of baicalein was able to improve the animal’s lifespan by up to 16.5% (Figure 4A). At the same concentration, chrysin, 7,8-dihydroxyflavone and 6-hydroxyflavone-treated worms had increases in the mean lifespan of 8.46% (Figure 4C), 4.9% (Figure 4F) and 2.36% (Figure 4D), respectively. The best result was obtained at 25 µM for baicalein with an increase of 18.49% in the animal’s longevity (Figure 4D). On the other hand, scutellarein (Figure 4B) and 6,7-dihydroxyflavone (Figure 4E) had small negative effects on the lifespans of treated worms, and high concentrations of 7,8-dihydroxyflavone also decreased the lifespan of *C. elegans* (Figure 4F).

### 3.4. Effect of Flavonoids as Gene Modulators in Longevity Pathways

Baicalein, chrysin and 6-hydroxyflavone showed the highest extensions of the lifespan, and at the same time were able to produce a significant decrease in fat storage in *C. elegans*. These two events are related, since they might be responses to the modulation of gene expression which depends on the IIS insulin signaling pathway via DAF-16 or depends on the redox active signaling pathway via SKN-1. In order to elucidate which pathways are involved in the effects of these three flavonoids, related mutant strains were employed at the optimal concentration obtained in the lifespan’s assay. Thus, 25 µM of baicalein, 10 µM of chrysin and 100 µM of 6-hydroxyflavone were employed to further investigate the pathways involved in their health-promoting effects in *C. elegans*. In addition, the basal effect of DMSO was taken into account, since a previous study reported that DMSO is able to modulate lifespan of the worms via DAF-16 [23].

#### 3.4.1. Effect of Flavonoids on the IIS Insulin Signaling Pathway

**Figure 5:**
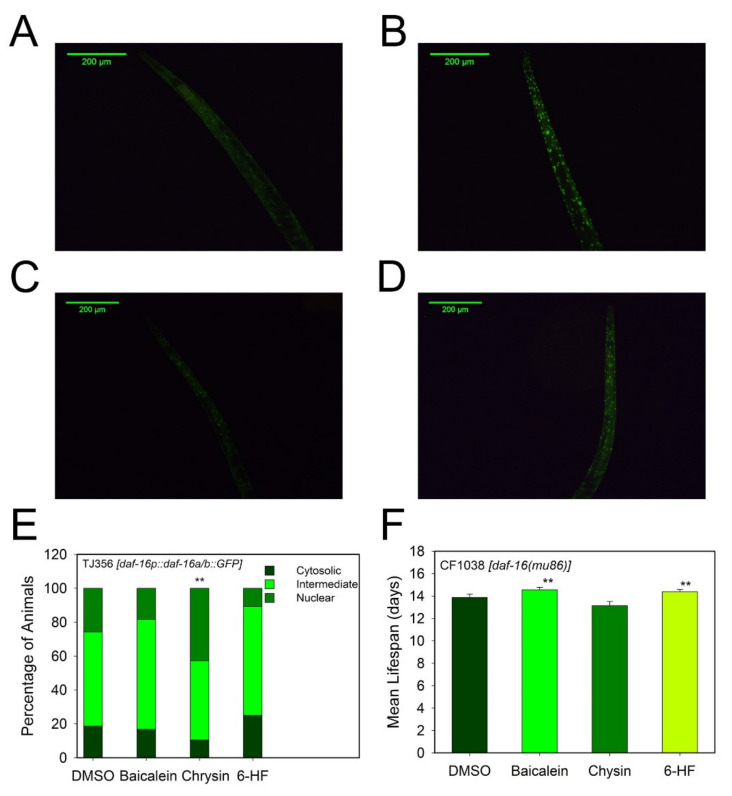
Biological confirmation of flavonoids’ activities in the insulin signaling pathway. Representative images are shown for (**A**) TJ356 (*daf-16::GFP*) negative control, (**B**) localization of TJ356 (*daf-16::GFP*) in worms treated with DMSO, (**C**) localization of TJ356 (*daf-16::GFP*) after baicalein treatment and (**D**) (*daf-16::GFP*) localization after chrysin treatment. (**E**) Histogram summarizing the fraction of TJ356 worms with cytosolic, intermediate or nuclear localization of DAF-16 (*daf-16::GFP*) after flavonoid treatment. (**F**) Mean lifespan of CF1038 (*daf-16 (mu86)*) worms treated with 25 µM of baicalein, 10 µM of chrysin or 100 µM of 6-hydroxyflavone. Data are represented as mean ± S.D, *n* > 10; two independent experiments were performed; ** significant at *p* ≤ 0.05 by ANOVA test. Scale bar: 200 μm, magnification 10×.

The effect of the flavonoids on the insulin signaling pathway was estimated in vivo with the daf-16 deficient strain CF1038 (*daf-16(mu86)*) and the fluorescent strain of *C. elegans* TJ356 (Figure 5). Chrysin effect on lifespan was lost in the mutant strain CF1038, where *daf-16* is not present, but baicalein and 6-hydroxyflavone increased the lifespan of the *daf-16*-deficient mutant strain (Figure 5F and Figure S4). These results were further supported by the fluorescent strain TJ356, which presents GFP fused with the transcription factor DAF-16 (Figure 5A–D). The results (Figure 5E) show that chrysin activated the translocation of DAF-16 to the cell nuclei with 58.6% of worms showing nuclear fluorescence (Figure 5D,E). Baicalein and 6-hydroxyflavone showed lesser effects on DAF-16 translocation than the dissolvent DMSO (16.1%) (Figure 5C,E).

#### 3.4.2. Effect of Flavonoids on the Redox Active Signaling Pathway

**Figure 6:**
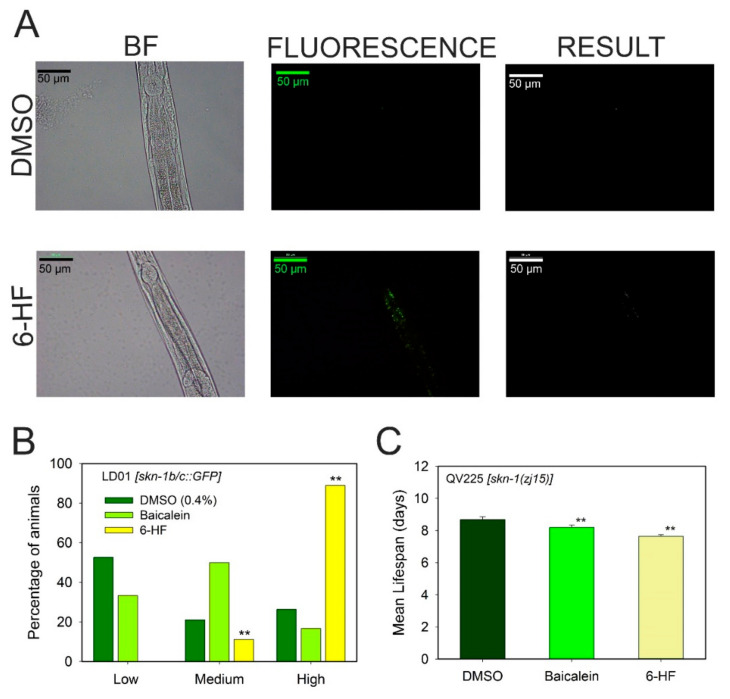
Effects of the different flavonoids on the redox active pathway. (**A**) LD1 strain (*skn-1 b/c::GFP*) treated with DMSO as control and treated with 100 µM of 6-hydroxyflavone. (**B**) Results of the integrated fluorescence of the gut in LD1 worms treated with 100 µM of each flavone. (**C**) Changes in mean lifespan results for mutant worms QV255 (*skn-1(zj15)*) treated with 100 µM of flavone. Data are represented as mean ± S.D, *n* > 10; two independent experiments were performed; ** significant at *p* ≤ 0.05 by ANOVA test. Scale bar: 50 μm, magnification 40×.

The possible effects of baicalein and 6-hydroxyflavone in vivo due to the redox active pathway were measured with the SKN-1-deficient strain QV225 (*skn-1(zj15)*) of *C. elegans* and the fluorescent strain LD1 [*skn-1 b/c::GFP*] (Figure 6). SKN-1 is the *C. elegans* ortholog to the human Nrf2, a transcription factor that regulates the phase II detoxification response to oxidative stress [24]. The Lifespan Machine showed that the extension effects produced on the lifespans of the wild-type worms treated with baicalein and 6-hydroxyflavone were lost in the *skn-1* deficient strain (Figure 6C and Figure S5). This suggests that the longevity effect of these flavones is dependent on the redox active pathway which involves SKN-1. To further investigate this hypothesis, the LD1 strain of *C. elegans* was used. The flavonoid 6-hydroxyflavone produced the translocation of the SKN-1 transcription factor to the nuclei in LD1 worms, whereas baicalein did not produce a significant translocation of the transcription factor to the gut cell nuclei (Figure 6B).

### 3.5. Baicalein Effect on C. elegans Gene Expression

RNA microarray assays from worms treated with baicalein were performed to investigate the possibility that the molecule could modulate *C. elegans* gene expression. The microarray data obtained from this analysis have been deposited in the GEO database (NCBI) [https://www.ncbi.nlm.nih.gov/geo/] and assigned the identifier GSE134775 (released on 11 March 2021). DMSO-treated worms were used as a control and their results were compared with water-treated results, to further characterize the dissolvent effects and compare them with those obtained from baicalein-treated worms. The DMSO microarray showed the upregulation of microbial defense and innate immune response genes *lys-7* (3.35-fold vs. control, *p* ≤ 0.05), *spp-1* (1.59-fold vs. control, *p* ≤ 0.05), *spp-2* (3.15-fold vs. control, *p* ≤ 0.05) and *spp-8* (3.78-fold vs. control, *p* ≤ 0.05).

**Figure 7:**
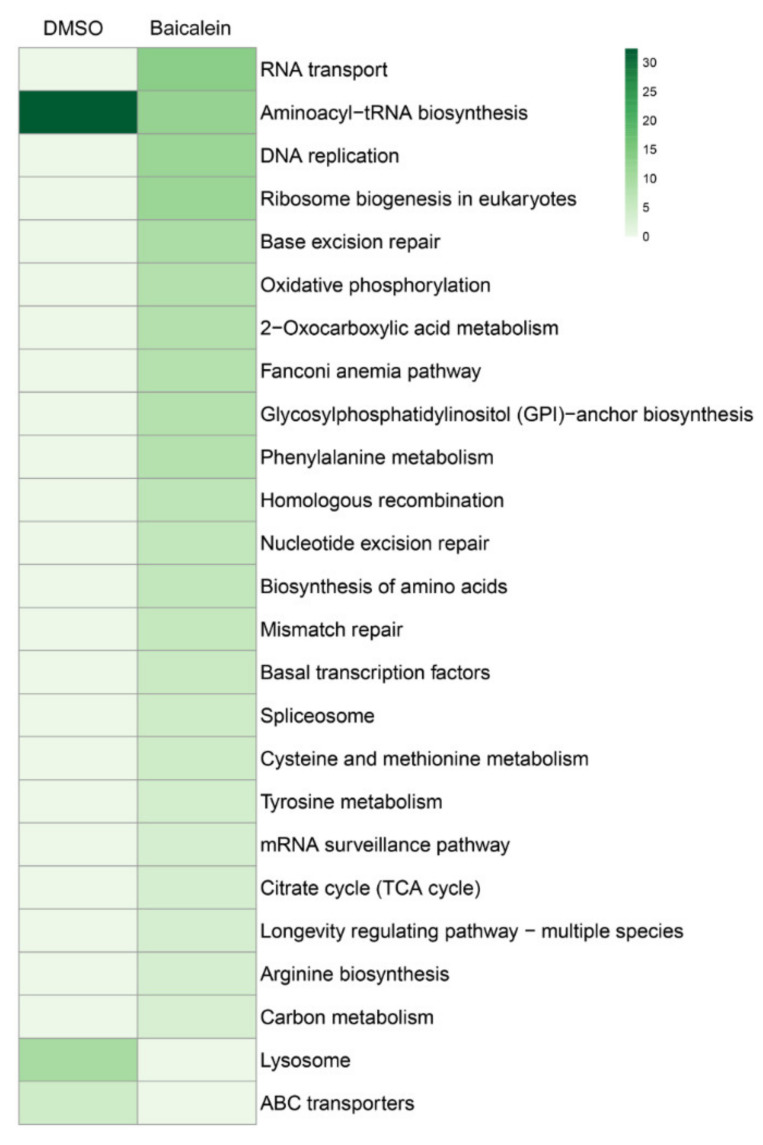
Principal component analysis (PCA) of *C. elegans* pathways altered by baicalein treatment (100 µM) vs. control worms treated with DMSO.

Microarray results for baicalein-treated worms indicated that several pathways were modulated, as illustrated in Figure 7 and in Table S2. The microarrays showed that baicalein treatment modulated the expression of genes *daf-16* (-1.33-fold vs. control, p ≤ 0.05), *sir 2.1* (−1.5-fold vs. control, *p* ≤ 0.05), *akt-1* (−1.58-fold vs. control, *p* ≤ 0.05), *age-1* (−2.1-fold vs. control, *p* ≤ 0.05), *ctl-2* (2.5-fold vs. control, *p* ≤ 0.05), *mtl-1* (6.7-fold vs. control, *p* ≤ 0.05 and *mtl-2* (8.8-fold vs. control, *p* ≤ 0.05) and *daf-18* (−5.55-fold vs. control, *p* ≤ 0.05). Baicalein also modulated the expression of mitochondrial proteins, such as the poly(ADP-ribose) polymerases (PARPs), *parp-1* (formerly known as *pme-1*, −4.14-fold vs. control, *p* ≤ 0.05) and *parp-2* (−3.8-fold vs. control, *p* ≤ 0.05).

mTOR (mammalian target of rapamycin) pathway was also affected by baicalein treatment. Microarrays showed downregulation of *let-363* (−1.26-fold vs. control, *p* ≤ 0.05), *daf-15* (−1.7-fold vs. control, *p* ≤ 0.05) and *ife-3* (−2.8-fold vs. control, *p* ≤ 0.05), *rict-1* (−2.00-fold vs. control, *p* ≤ 0.05), and overexpression of *pha-4* (1.03-fold vs. control, *p* ≤ 0.05).

## 4. Discussion

All organisms generate heat shock proteins (HSPs) as a means of protection from oxidative damage. This response could be enhanced by the addition of bioactive compounds which confer resistance to oxidative stress to cells and organisms. However, the results obtained from the strain TJ375 showed that only 6,7-dihydroxyflavone and chrysin had a protective effect related to oxidative stress, by decreasing the fluorescence by up to 88.9% and 22.5%, respectively. The results from the treatment with 6,7-dihydroxyflavone suggest that its ability as stress-response gene modulator was stronger than previously tested polyphenols such as aspalathin or epigallocatechin gallate. These compounds increased the oxidative stress resistance of the treated *C. elegans* by 27.0% and 26.74% respectively [25,26]. The rest of the flavonoids here tested showed little activity towards oxidative stress in the worms (Figure 2B,D), suggesting that even through structurally similar, their effects, stability or bioavailability may be different. To further study the effect of flavonoids on oxidative stress resistance, the expression of the enzymes GST-4 and GCS-1 was analyzed. GCS-1 is regulated by SKN-1 transcription factor, while GST-4 could be regulated in a *skn-1*-dependent manner, or as demonstrated by Detienne et al. [27], the activation can be mediated by the epidermal growth factor (EGF) pathway and the EOR-1 transcription factor. This is independent of SKN-1 and the oxidative stress resistance pathway. All the flavones studied in this work showed effects towards both of the reporters except chrysin, which only showed a mild effect with the GST-4 reporter and scutellarein, which did not have any effect in the tested *C. elegans* strains (Figure S1E,F).

The bioactive properties of flavonoids are also related to fat metabolism and consequent reductions of fat storage and obesity [28,29]—a situation that increases the risk of suffering from several pathologies that nowadays affect 11–15% of the worldwide population [30], including cardiovascular diseases, type 2 diabetes and cancer. The results obtained after ORO staining showed that the six molecules studied were able to reduce the fat storage. These results agree with those reported from other flavonoids, such as naringenin and kaempferol [31], which eliminated 60–80% of total fat storage, and it is an indication of the ability of these compounds to improve the health by reducing fat accumulation.

The health-promoting effects of flavonoids in the diet were also measured as their ability to expand the lifespan of the wild-type *C. elegans*. The obtained survival curves and mean lifespans indicated that the flavonoids tested expanded the *C. elegans* mean lifespan in a dose-dependent manner over the concentration ranges used (Figure 3, Figure S3 and Table S1). Prior studies have shown similar results with other flavonoids, such as myricetin and quercetin, which prolonged *C. elegans’* lifespan by 18.0% and a 5.8%, respectively, when worms were treated with 100 µM of each compound [11]. The researchers associated this lifespan extension effect with the number of hydroxyl groups in the B-ring and with the structure of the C-ring, being the most active molecule myricetin, which presents three hydroxyl groups in the B-ring. According to this hypothesis, 6-hydroxyflavone should be the molecule with the lowest antioxidant activity, and therefore less active, since it presents only one hydroxyl group. However, with only one hydroxyl group it was probably the most stable and the least prone to forming oxidation species that may could the bioactive potential of the molecules when exposed to the medium for 48 h. Scutellarein, having four hydroxyl groups, and 6,7-dihydroxyflavone and 7,8-dihydroxyflavone, with two hydroxyl groups in ortho positions, are more susceptible to oxidation than 6-hydroxyflavone, with one hydroxyl group, chrysin, with two hydroxyls in meta positions, and baicalein, with three hydroxyl groups in ortho positions. Nevertheless, the assay revealed the potential of all the tested flavonoids as health-promoting compounds, mainly baicalein, chrysin and 6-hydroxyflavone.

The loss of chrysin’s effects in the mutant strain CF1038 (*daf-16(mu86)*) showed that its positive effects are related to the IIS insulin signaling and strictly dependent on DAF-16. The transcription factor DAF-16 is active when it is translocated into the cell nuclei, and it is inactive when it shows low diffuse fluorescence in the cytoplasm. Thus, the translocation of DAF-16 to the nuclei observed in the mutant strain TJ356 (*daf-16::GFP*) supported that chrysin prolongs *C. elegans’* lifespan in a DAF-16-dependent manner.

Baicalein and 6-hydroxyflavone did not produce similar results, suggesting that they are not dependent on DAF-16 and their health-promoting effects might be due to the redox active signaling pathway. In *C. elegans*, this pathway involves the transcription factor SKN-1, an ortholog to the human Nrf2 [24]. SKN-1 activates enzymes that work as radical scavengers and transfer glutathione. SKN-1 is located in the ASI neurons (putative hypothalamus) in response to dietary restrictions, and in the gut nuclei in response to oxidative stress.

The results obtained from the SKN-1 deficient strain QV225 (*skn-312 1(zj15)*) of *C. elegans* clearly indicated for the first time that the longevity effects of 6-hydroxyflavone in *C. elegans* are mediated by the SKN-1 transcription factor and the redox active pathway. These results were supported by those obtained from the mutant strain LD1 (*skn-1 b/c::GFP*), showing green fluorescence in the gut cell nuclei and ASI neurons when the transcription factor was active (Figure 5A,B). Moreover, 6-hydroxyflavone increased the expression of two downstream targets of SKN-1: GCS-1 and GST-4 (Figure S1), supporting once again that the beneficial effects of this compound are mediated by the redox active pathway.

However, lifespan expansion effects of baicalein were not totally dependent on SKN-1 (although it upregulated GST-4 and GCS-1 expression), or totally dependent on the IIS insulin signaling pathway, and further investigation at the transcriptomic level was necessary to elucidate the mechanisms involved in its lifespan extension.

Microarray analysis of worms treated with DMSO as the control showed the upregulation of microbial defense and innate immune response genes such as *lys-7* that encode antimicrobial lysozymes or the SPP family (Saposin-like protein family), along with *spp-1*, *spp-2* or *spp-8*, which are antibacterial peptides present in the lumen of *C. elegans* [32]. Overexpression of *lys-7* by DMSO has been previously reported using quantitative real-time PCR [23], supporting the results obtained in this microarray.

Baicalein treatment downregulated genes involved in the insulin signaling pathway, including *daf-16*, *sir 2.1*, *akt-1* and *age-1*. These results are consistent with the results obtained with the DAF-16-deficient mutant strain and the above-mentioned TJ375 strain, which was used to evaluate the antioxidant effects of the compounds in vivo. Although baicalein is not DAF-16-dependent, the molecule is able to upregulate the genetic expression of some enzymes downstream of DAF-2/DAF-16 involved in stress resistance, such as catalase *ctl-2* and metallothioneins *mtl-1* and *mtl-2*; and elevated levels of these proteins may contribute to ROS degradation and therefore to lifespan extension [33]. Downregulation of mitochondrial *parp-1* and *parp-2* could be involved in the increase of lifespan produced by baicalein. It has been reported that RNAi silencing of *parp-1* or treatment with PARP inhibitors in wild-type worms increased their lifespan in the range of 15–29% by raising the NAD+ levels [34]. In addition, several studies concluded that the repression of *parp-1* could be an interesting approach to develop synthetic lethality in cancer cells [35] and in the regulation of the anti-inflammatory response [36].

Baicalein also inhibited the mTOR pathway by downregulating *let-363*, the *C. elegans* ortholog to human mTOR; *daf-15*, the ortholog to human RPTOR; *ife-3*; and *rict-1;* and resulted in the overexpression *pha-4*. Many studies have reported relationships between mTOR and human diseases such as cancer, cardiovascular diseases, diabetes, obesity and neurological disorders, which are involved in age-related diseases and in lifespan regulation [37]. mTOR is the main component of two different multiprotein complexes mTORC1 and mTORC2, which are involved in the regulation of the synthesis of proteins needed for cell growth and proliferation [38]. It has been reported that an inhibition of mTORC1 or mTORC2 in *C. elegans* produced a lifespan extension dependent on SKN-1, but the inhibition of mTOR did not increase the presence of SKN-1 in cell nuclei [24,39], as happened to the worms treated with baicalein in this study. In addition, PHA-4 transcription factor is described as necessary for the longevity effects associated with mTOR inhibition [40], and indeed *pha-4* was upregulated in the worms treated with baicalein, as shown in the microarrays performed.

Microarray testing was performed for worms treated with baicalein at the highest concentration measured (i.e., 100 µM) and the results show how even though the lifespan extension at 100 µM was low, the effects of the flavonoid baicalein on *C. elegans* may be partially related to the downregulation of the expression of mTOR, which produces a lifespan extension dependent on SKN-1, this being the same effect reported in worms treated with rapamycin [39]. These results agree with the mechanism of action proposed for baicalein in human cancer cell lines [41,42], which showed growth inhibition of cancer cells via mTOR inhibition. Further studies with experiments at lower concentrations of baicalein would be of relevance to link the regulation of these genes and the total extension of *C. elegans* lifespan. From the results of the present study, it is clearly shown that the lifespan extension of *C. elegans* and human cancer cells’ inhibition by baicalein share the same molecular mechanism.

Baicalein seems also to be a repressor of DAF-18, the ortholog of human PTEN tumor suppressor, which is involved in several diseases, such as Bannayan–Riley–Ruvalcaba syndrome, carcinomas (multiple) and reproductive organ cancer (multiple). It is proposed that a reduction of PTEN may increase insulin and/or insulin-like growth factor signaling. However, an increase of PTEN activity may cause insulin resistance, as happens in late onset diabetes disease [43]. In *C. elegans age-1* mutants, the suppression of *daf-18* caused a decrease in fat storage [43], and adipogenesis reduction has also been reported as a response of mTOR and RPTOR inhibition [44].

Thus, the fat storage reduction reported in this work by the worms treated with baicalein (Figure 2) agrees with the inhibition of mTOR and RPTOR and the additional activation of the redox active pathway and SKN-1 transcription factor. Besides, the downregulation of PARP expression may increase the NAD^+^ levels and therefore contribute to the lifespan extension.

## 5. Conclusions

The results showed that all flavonoids tested increased the mean lifespan of the model animal *C. elegans* in a dose-dependent manner, the most active molecules being baicalein, chrysin and 6-hydroxyflavone. The cytoplasmic stress resistance assay showed that only 6,7-dihydroxyflavone was able to enhance the oxidative stress resistance of *C. elegans* by reducing the pharynx fluorescence by 88.9%. However, the oxidative stress resistance assay showed that 6-hydroxyflavone and 6,7-dihydroxyflavone increased the stress resistance by upregulating the GST-4 expression by 65.3% and 94.3% respectively. The other flavonoids had mild effects, suggesting that their positive effects on lifespan are not mainly related to oxidative stress resistance and radical scavenging properties. The results from the mutant strains for DAF-16 and SKN-1 transcription factors treated with the best enhancers of lifespan showed how different pathways are involved in the flavonoids’ lifespan extensions of *C. elegans*.

Chrysin expanded the lifespans of the worms in a DAF-16-dependent manner, whereas baicalein and 6-hydroxyflavone prolonged *C. elegans’* lifespan via SKN-1 pathway. RNA microarray for baicalein-treated worms showed the downregulation of the mTOR pathway, which leads to a reduction in lipids and to an activation of the redox active pathway and SKN-1 transcription factor, thereby being responsible for the lifespan extension in *C. elegans*. Additionally, the repression of some mitochondrial protein’s genes such as *parp-1* and *parp-2* may contribute to the longevity effects shown. Altogether, the results are an indication that baicalein may be a potential drug for the treatment of diseases in which mTOR or PARPs are involved. Additionally, the tested flavonoids should be further investigated for possible effects in the prevention and treatment of obesity, as they reduced fat accumulation in the model animal. In this sense, the most effective molecule was chrysin, which was able to reduce the fat storage by 38.9% compared to control animals.

## Data Availability

The microarray data from this publication have been deposited in the GEO database (NCBI) (https://www.ncbi.nlm.nih.gov/geo/ released and accessed on 11 March 2021) and assigned the identifier GSE134775.

## Supplementary Materials

The following are available online at https://www.mdpi.com/2076-3921/10/3/438/s1. Figure S1. Flavones’ effects on GST-4 and GSC-1 expression, Figure S2. Effects of flavonoids stained with Red O (ORO), Figure S3: Lifespan curves adjusted to Kaplan–Meier estimator for the *C. elegans* strain N2 treated with individual flavonoids, Figure S4: Lifespan curves adjusted to Kaplan–Meier estimator for the *C. elegans* strain CF1038 treated with individual flavonoids baicalein, chrysin and 6-hydroxyflavone, Figure S5: Lifespan curves adjusted to Kaplan–Meier estimator for the *C. elegans* strain QV225 treated with baicalein and 6-hydroxyflavone, Table S1: Survival data for the *C. elegans* treated with flavonoids. Table S2. Pathways modulated by baicalein treatment.
