# Supplementary material for: Flavonoids’ Effects on Caenorhabditis elegans’ Longevity, Fat Accumulation, Stress Resistance and Gene Modulation Involve mTOR, SKN-1 and DAF-16
Source: Antioxidants (Basel). 2021 Mar 12;10(3):438. doi: 10.3390/antiox10030438 (PMC8001597; doi:10.3390/antiox10030438)
Supplement: Supplementary file 1 [file antioxidants-10-00438-s001.pdf]

Supplementary material

Flavonoids' Effects on Caenorhabditis elegans' Longevity, Fat Accumulation, Stress Resistance and Gene Modulation Involve mTOR, SKN-1 and DAF-16

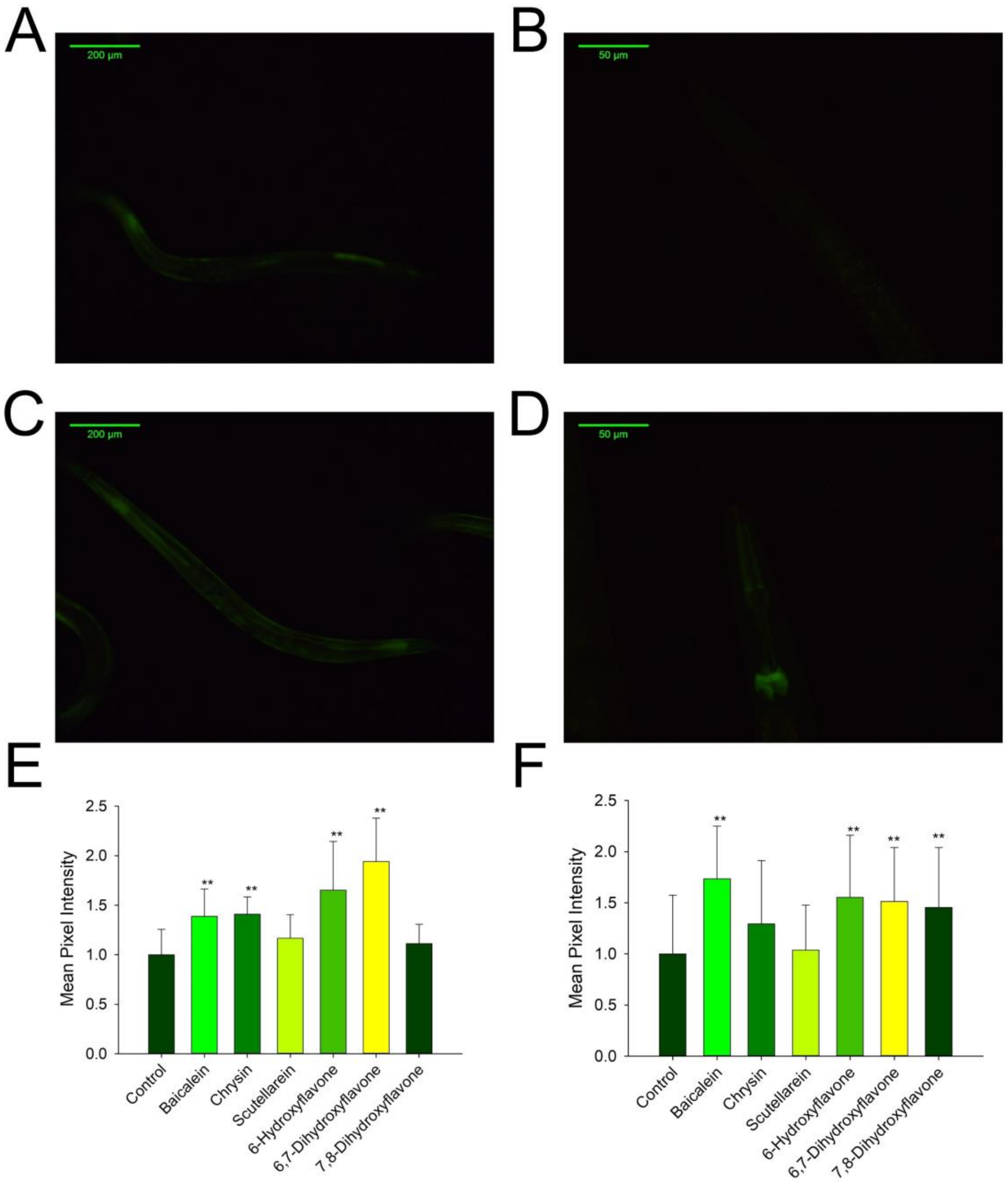

Figure S1. Flavones effect on GST-4 and GSC-1 expression. (a-d) Representative image of: (a) Control *C. elegans* of the CL2166 (GST-4 reporter) strain. (b) Control animal of the LD1171 (GCS-1 reporter) strain. (c) CL2166 worm treated with 6,7-dihydroxyflavone (100 µM). (d) *C. elegans* LD1171 treated with baicalein (100 µM). Scale bar for (a) and (c) 200 µm, magnification 10x, and scale bar for (b) and (d) 50 µm, magnification 40x. (e) Quantification of the flavones effect on the expression of GST-4. (f) Quantification of the flavones effect on the expression of GSC-1. Data is represented as mean±S.D, n > 10, two independent experiments were performed, **significantly at $p \leq 0.05$ by ANOVA test.

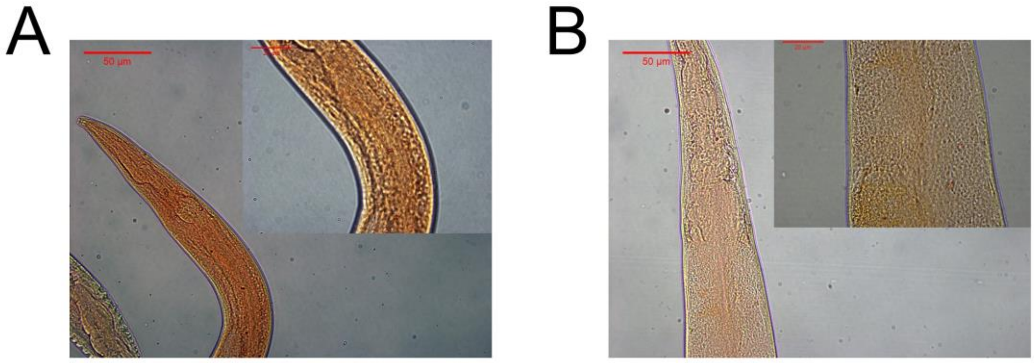

Figure S2. Effect of flavonoids stained with Red O (ORO). **(a)** DMSO control worm magnification 40x, inset magnification 100x. **(b)** Worm treated with 100 µM of chrysin magnification 40x, inset magnification 100x. Scale bar: 50 µm, scale bar of the insets 20 µm.

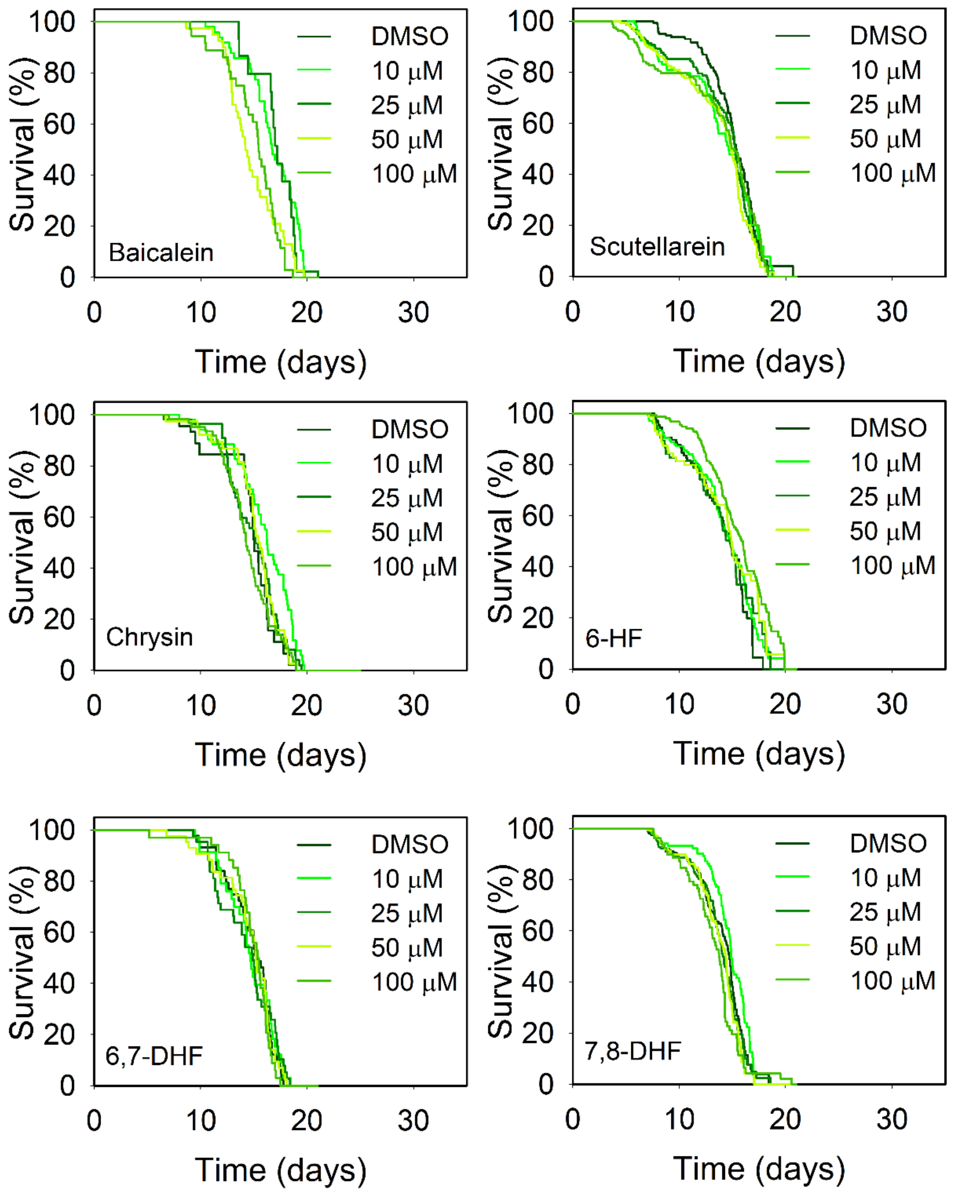

Figure S3. Lifespan curves adjusted to Kaplan-Meier estimator for the *C. elegans* strain N2 treated with individual flavonoids.

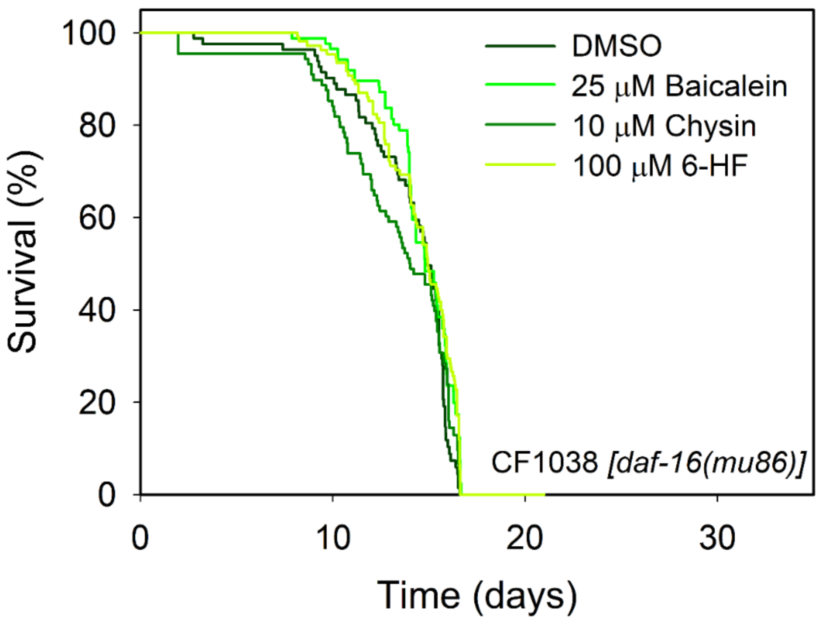

Figure S4. Lifespan curves adjusted to Kaplan-Meier estimator for the *C. elegans* strain CF1038 treated with individual flavonoids baicalein, chrysin and 6-hydroxyflavone.

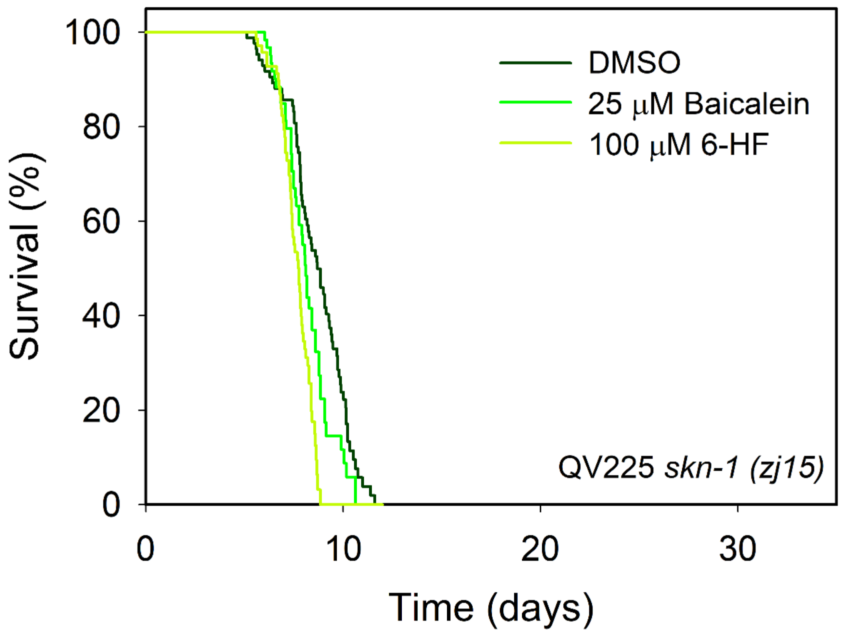

Figure S5. Lifespan curves adjusted to Kaplan-Meier estimator for the *C. elegans* strain QV225 treated with baicalein and 6-hydroxyflavone.

Supplemental Table 1. Survival data for the *C. elegans* treated with flavonoids

C. elegans Strain	Flavonoid	Concentration (μM)	n	Mean Lifespan (days)	S. E (±day)	Lifespan Change (%)	*p*-value *vs* Control
N2 Wild-type	Baicalein	0	166	14.33	0.24		
		10	98	16.7	0.25	16.54	0**
		25	95	16.98	0.27	18.49	0**
		50	80	14.72	0.28	2.72	0.0576
		100	72	14.96	0.29	4.40	0.0077**
	Chrysin	0	90	14.66	0.28		
		10	104	15.9	0.28	8.46	0.000006**
		25	110	14.92	0.25	1.77	0.1914
		50	76	15.1	0.29	3.00	0.226
		100	124	14.38	0.23	-1.91	0.4426
	Scutellarein	0	163	14.58	0.29		
		10	109	13.79	0.4	-5.42	0.3076
		25	105	14.08	0.31	-3.43	0.3288
		50	136	13.54	0.32	-7.13	0.0457**
		100	123	13.59	0.36	-6.79	0.3814
	7,8-Dihydroxyflavone	0	175	13.89	0.21		
		10	105	14.58	0.23	4.97	0.0263**
		25	160	13.71	0.22	-1.30	0.4389
		50	156	13.59	0.2	-2.16	0.1045
		100	83	13.24	0.31	-4.68	0.0177
	6,7-Dihydroxyflavone	0	88	14.71	0.24		
		10	70	14.55	0.3	-1.09	0.4585
		25	86	14.29	0.29	-2.86	0.5925
		50	86	14.52	0.28	-1.29	0.8729
		100	88	14.77	0.27	0.41	0.2216
	6-Hydroxyflavone	0	111	13.96	0.29		
		10	140	14.29	0.3	2.36	0.3262
		25	88	14.04	0.4	0.57	0.5242

		50	74	14.41	0.45	3.22	0.1312
		100	159	15.61	0.25	11.82	0.0001**
(skn-1(zj15))		0	82	13.87	0.31		
	Baicalein	25	86	14.56	0.21	4.97	0.0168**
	Chrysin	10	89	13.15	0.37	-5.19	0.4943
	6-Hydroxyflavone	100	108	14.38	0.21	3.68	0.0079**
CF1038 *(daf-16(mu86))*		0	85	8.68	0.17		
	Baicalein	25	61	8.19	0.16	-5.65	0.0172**
	6-Hydroxyflavone	100	70	7.65	0.1	-11.87	7.8e-8**

Supplemental Table 2. Pathways modulated by baicalein treatment. Data obtained from the RNA microarray results vs DMSO control worms.

Pathway Name	DMSO			Baicalein		
	Enrichment Score	Enrichment *p*-value	% genes	Enrichment Score	Enrichment *p*-value	% genes
RNA transport	0.00	0.00	0.00	13.31	1.66E-06	60.00
Aminoacyl-tRNA biosynthesis	32.42	8.30E-15	52.94	12.05	5.84E-06	70.59
DNA replication	0.00	0.00	0.00	11.64	8.78E-06	78.26
Ribosome biogenesis in eukaryotes	0.00	0.00	0.00	11.49	1.03E-05	76.00
Base excision repair	0.00	0.00	0.00	9.16	1.05E-04	90.91
Oxidative phosphorylation	0.00	0.00	0.00	8.31	2.45E-04	56.60
2-Oxocarboxylic acid metabolism	0.00	0.00	0.00	8.12	2.96E-04	90.00
Fanconi anemia pathway	0.00	0.00	0.00	8.10	3.03E-04	71.43
Glycosylphosphatidylinositol (GPI)-anchor biosynthesis	0.00	0.00	0.00	7.84	3.93E-04	140.00
Phenylalanine metabolism	0.00	0.00	0.00	7.83	3.97E-04	100.00
Homologous recombination	0.00	0.00	0.00	6.52	1.48E-03	73.33
Nucleotide excision repair	0.00	0.00	0.00	6.09	2.28E-03	61.54
Biosynthesis of amino acids	0.00	0.00	0.00	5.92	2.69E-03	53.19
Mismatch repair	0.00	0.00	0.00	5.69	3.39E-03	71.43
Basal transcription factors	0.00	0.00	0.00	5.09	6.13E-03	63.16
Spliceosome	0.00	0.00	0.00	4.52	1.09E-02	45.95
Cysteine and methionine metabolism	0.00	0.00	0.00	4.45	1.16E-02	55.56
Tyrosine metabolism	0.00	0.00	0.00	3.74	2.37E-02	58.82
mRNA surveillance pathway	0.00	0.00	0.00	3.56	2.86E-02	46.15
Citrate cycle (TCA cycle)	0.00	0.00	0.00	3.47	3.11E-02	50.00
Longevity regulating pathway - multiple species	0.00	0.00	0.00	3.31	3.64E-02	48.57
Arginine biosynthesis	0.00	0.00	0.00	3.25	3.87E-02	66.67
Carbon metabolism	0.00	0.00	0.00	3.01	4.91E-02	42.47
Lysosome	10.01	4.49E-05	18.67	0.00	0.00	0.00
ABC transporters	4.48	0.01	20.00	0.00	0.00	0.00
